# Gait Characteristics Related to Fatigue in Patients With Type 2 Diabetes: Cross-Sectional Study

**DOI:** 10.7759/cureus.87487

**Published:** 2025-07-07

**Authors:** Daisuke Iwaki, Makoto Takahashi, Toshihiro Kawae, Yuki Nakashima, Kenichi Fudeyasu, Kazutoshi Iba, Kai Ushio, Yukio Mikami

**Affiliations:** 1 Department of Clinical Practice and Support, Hiroshima University Hospital, Hiroshima, JPN; 2 Department of Biomechanics, Hiroshima University, Hiroshima, JPN; 3 Department of Physical Therapy, Tohto University, Chiba, JPN; 4 Department of Rehabilitation Medicine, Osaka Saiseikai Ibaraki Hospital, Osaka, JPN; 5 Department of Rehabilitation, Hiroshima General Hospital, Hiroshima, JPN; 6 Department of Rehabilitation Medicine, Hiroshima University Hospital, Hiroshima, JPN

**Keywords:** acceleration, diabetic peripheral neuropathy, fatigue, gait, type 2 diabetes

## Abstract

Aim

This study aimed to determine the gait abnormalities associated with fatigue in patients with type 2 diabetes. Although walking is widely recommended as an effective form of exercise for diabetes management, many patients experience difficulty maintaining exercise routines due to fatigue. We hypothesized that patients with type 2 diabetes who experience fatigue during exercise would exhibit gait abnormalities that contribute to fatigue, such as abnormal increases or decreases in gait acceleration, particularly reduced regularity in vertical acceleration.

Methods

In this cross-sectional study, we recruited 50 patients with type 2 diabetes mellitus (mean age: 64.9 ± 12.0 years; BMI: 25.3 ± 4.6 kg/m²; 32 males and 18 females; HbA1c: 10.0 ± 2.2%) who were admitted to two hospitals in Japan for glycemic control between April 2020 and March 2021. Gait parameters were measured using a triaxial accelerometer during a 16-meter free walking test.

Results

Participants were divided into fatigue (n = 29; age: 60.7 ± 12.4 years; BMI: 26.4 ± 4.8 kg/m²) and no-fatigue (n=21; age: 70.8 ± 9.1 years; BMI: 23.8 ± 4.0 kg/m²) groups based on the Exercise Benefits and Barriers Scale.

No significant differences were observed in the root mean square (RMS) or RMS ratio between the groups. Statistical analyses were conducted following normality confirmation using the Shapiro-Wilk test. The unpaired t-test and Wilcoxon signed-rank test were applied to normally and non-normally distributed data, respectively, and categorical variables were compared using the chi-square test. Multivariate logistic regression analysis was also performed. The vertical autocorrelation coefficient (ACVT), representing gait regularity, was significantly lower in the fatigue group compared to the no-fatigue group (p=0.044; CI: −0.05 to 0.00). Activity energy expenditure (AEE), calculated based on acceleration, BMI, and sex as a proxy for oxygen consumption, was significantly higher in the fatigue group (p=0.039; CI: 0.03 to 0.45). Multivariate analysis identified age (p=0.008; OR=0.916; CI: 0.84 to 0.98) and ACVT (p=0.038; OR=0.16; CI: 0.02 to 0.90) as significant factors.

Conclusion

These findings suggest that lower regularity of vertical acceleration during walking in patients with type 2 diabetes is a factor causing fatigue and may be a barrier to exercise continuation.

## Introduction

The role of diabetes management is to maintain blood glucose levels within a target range through integrated approaches, including medication adherence, regular blood glucose monitoring, appropriate diet, physical activity, and patient education [1]. Exercise is strongly recommended for the treatment of diabetes mellitus. However, several patients find it difficult to continue exercising because of their physical condition, time, and weather conditions [2]. The aforementioned factors also apply to healthy people; however, increased fatigue during exercise with decreased physical function may additionally affect the persistence of exercise in patients with diabetes [3].

Fatigue can be characterized as peripheral or central. Peripheral fatigue, such as that observed in skeletal muscles, results from a combination of neurological, musculoskeletal, and metabolic abnormalities, including decreased glycogen storage in the liver or muscle, decreased oxygen consumption during activity, and changes in muscle fibers due to physical inactivity or aging [4]. Recent evidence indicates that muscle fatigability may be intensified in type 2 diabetes [5].

Muscle fatigability is an important component of neuromuscular performance and is fundamental to several activities of daily living that require repeated or sustained contractions. Although walking is a daily activity that requires repetitive contractions performed by all patients with diabetes, studies have reported that numerous walking parameters are altered in patients with diabetes [6]. Patients with diabetes prefer to walk at a reduced self-selected comfortable speed compared with age-matched healthy subjects [7]. This slower gait is associated with shorter step lengths and longer stance phases with more co-contractions of the agonist and antagonist muscles, which may contribute to the increased lower extremity fatigue in the walking motion [8]. These findings suggest that the slower gait speed in patients with diabetes represents an active strategy to reduce the relative effort. In contrast, a recent study using accelerometers also reported increased center-of-gravity acceleration during walking in patients with type 2 diabetes, and this greater displacement of center-of-gravity acceleration increases oxygen consumption [9]. Activity-related energy expenditure for the same exercise is higher in diabetic patients than in healthy subjects [10]. Thus, the altered gait patterns and styles may increase physiological relative effort and energy expenditure, which in turn induce fatigue during walking in patients with type 2 diabetes. However, the gait characteristics related to fatigue during walking remain unclear in patients with type 2 diabetes. Thus, investigating gait abnormalities associated with fatigue in patients with diabetes may help develop strategies for gait modification, which in turn promote the improvement of gait fatigue and increase the continuity of exercise.

Gait characteristics in patients with diabetes can be measured using accelerometers, as reported in a recent systematic review [6]. The root-mean-square (RMS) of trunk acceleration, which represents amplitudes or variations, is frequently observed in gait analysis research [11]. Because gait is a continuous, cyclic, and repetitive activity, examining the overall magnitude of variability, such as RMS, and the regularity, which is one of the primary features of efficient gait, is useful. Indeed, a recent study examining regularity assessed using autocorrelation analysis showed significantly higher regularity values during gait in trained subjects than in untrained subjects [12]. This finding indicates that exercise training is associated with a more precise control of gait, thereby contributing to the maximum cost efficiency of gait and minimal metabolic energy. Thus, low regularity could reflect energetically wasteful conditions and gait characteristics related to fatigue during walking in patients with type 2 diabetes.

Previous studies have examined fatigue and gait in healthy elderly subjects and gait characteristics after experimentally inducing fatigue in type 2 diabetes patients [11,13,14]. These existing studies mostly focus on experimentally induced fatigue. These types of experiments only provide evidence regarding the effects of fatigue on gait characteristics. Therefore, research is needed to investigate the natural association between gait patterns and fatigue in the non-fatigued state in patients with type 2 diabetes. However, studies examining gait abnormalities associated with naturally occurring fatigue in type 2 diabetes are scarce. Thus, we compared gait parameters obtained using a triaxial accelerometer between the fatigue and no-fatigue groups based on the Exercise Benefits and Barriers Scale (EBBS) question "Does exercise make you tired?" [15]. This study aimed to determine the gait abnormalities associated with fatigue in patients with type 2 diabetes. We hypothesized that a group of patients with type 2 diabetes who experience fatigue during exercise would exhibit gait abnormalities that contribute to fatigue, such as abnormal increases or decreases in gait acceleration, particularly reduced regularity in vertical acceleration.

## Materials and methods

Participants

In this cross-sectional study, we recruited 50 patients with type 2 diabetes mellitus aged >40 years who were admitted to two hospitals in Japan between April 2020 and March 2021 for glycemic control. The ICD classification of the disease was E11 type 2 diabetes mellitus. The diagnosis of diabetes was made by an experienced physician with at least 10 years of clinical experience. Patients with central nervous system and orthopedic diseases and obvious cognitive decline or who have been diagnosed with dementia that affected walking were excluded. This study was approved by the Epidemiological Research Ethics Review Committee of Hiroshima University (approval number: E-1858). All participants received an explanation regarding the study protocol and provided informed consent.

Assessment of diabetes complications

Diabetic retinopathy and nephropathy were scored as follows. For diabetic retinopathy, "none" was scored as 0, whereas "simple retinopathy or more severe retinopathy" was scored as 1. Patients with diabetic nephropathy stages 1, 2, 3, and 4 were diagnosed with pre-nephropathy, incipient nephropathy, overt nephropathy, and renal failure. Among patients with diabetic nephropathy, those with stage <3 were scored as 0, whereas those with stage ≥3 were scored as 1 [16]. The presence of diabetic peripheral neuropathy (DPN) was confirmed using the Michigan Neuropathy Screening Instrument (MNSI), with scores of ≥2 indicating DPN, whereas scores of ≤1 indicated no DPN [17].

Perceived benefits and barriers scale

The EBBS contains 20 statements regarding perceived benefits and barriers to exercise and is scored using a five response, forced-choice Likert scale ranging from 1 (strongly disagree) to 5 (strongly agree). The perceived benefits and barriers scale, which includes five benefit subscales (i.e., physical benefit, psychological benefit, social benefit, weight management, and self-improvement) with 10 items and five barrier subscales (i.e., discomfort, lack of motivation, lack of time, lack of social support, and poor physical environment) with 10 items, was assessed using a self-administered questionnaire [15].

Physical function and performance

The maximal knee extensor force (KEF) was measured using a handheld dynamometer (µTas F-1; Anima Inc., Tokyo, Japan). The patients were seated at the end of the bed during measurements, after which isometric KEF was measured with the upper ankle joint fixed to the bed with a belt in a 90° hip joint and 90° knee joint posture, and the upper limbs were crossed in front of the body. Measurements were obtained twice on each side, and the average of the left and right maximum forces was used as the KEF, which was then divided by the body weight to obtain the weight-specific knee extensor strength (KEF/kg)[16]. Handgrip strength was measured using the Jamar-type handgrip meter. The subjects were instructed to sit on the edge of the bed and exert maximum force with the elbow joint at 90°. Measurements were obtained twice, once on each side, after which the average of the maximum forces on both sides was adopted as the representative value. The skeletal muscle index (SMI) was assessed using an InBodyS10 analyzer (InBody Co., Ltd., Seoul, Korea). The limbs were measured at the edge sitting position, and a five-minute break was taken before the measurement. The SMI was calculated by dividing the limb muscle mass by the square of the height.

Gait assessment using an accelerometer

One inertial sensor-embedded smart device (iPod Touch 7th generation, iOS operating system version 13.6.1; Apple, USA) with a measurement range of 72 g and 16-bit data output was used for the measurements. The global axes were defined as follows: positive X, Y, and Z values denoted anterior acceleration, right acceleration, and upward acceleration, respectively. The smart device was attached over the L3 spinous process using a Velcro™ belt to measure acceleration, and the subjects were instructed to walk along a 16 m straight path. Before each measurement, the subjects were kept in a standing position for approximately 10 s to conduct revisions and calibration of the accelerometer gravitation. The acceleration was sampled at 100 Hz. The walking speed was measured as the time taken to walk 10 m, excluding the acceleration and deceleration phases, each comprising 3 m. The test was conducted twice, and the acceleration data was the average of the two tests. The patients were requested to walk as usual.

Acceleration data analysis

The first and last two steps of the 16-m free walk were excluded from the analysis. A second-order Butterworth filter, supplied with forward and backward infinite impulse response band-pass coefficients (cutoff frequency, 0.5-25 Hz), was applied to reduce drift and integration errors. All signal processing was performed using custom-made software designed in MATLAB R2020a (MathWorks, Natick, MA, USA).

The RMS acceleration was calculated at the L3 spinous process in the vertical (VT), medial-lateral (ML), and anterior-posterior (AP) directions. Although the RMS has been used as a gait analysis index in various studies, it is affected by walking speed. Therefore, the walking speed should be kept constant, which is challenging on a normal walking path. Recently, an index called root mean square ratio (RMSR) has been attracting attention. The RMSR expresses the RMS of each direction as a ratio as follows: 1) $RMS_T = \sqrt{RMS_{AP}\,^2 + RMS_{ML}\,^2 + RMS_{VT}\,^2}$; 2) $RMSR_X = \frac{RMS_X}{RMS_T} \text{ where x represents the direction of acceleration.}$

Reports have shown that the RMSR provides information on gait abnormality while considering differences in individual walking speed and that the system can detect abnormal gait in patients with hip osteoarthritis and ataxia [18].

AC was calculated as the correlation with the acceleration shifted by one gait cycle. The harmonic ratio (HR) was computed using a digital Fourier transformation in each direction individually.

Activity energy expenditure (AEE) was calculated from the vector magnitude calculated from the acceleration of each axis, BMI, status, and sex, as follows: 3) $VM = \frac{1}{N}\sum_{i=0}^{N-1}\sqrt{a(i)_x^2 + a(i)_y^2 + a(i)_z^2}$; ​​​​​​​4) $AEE = -0.818 + 0.53 \times VM + 0.066 \times BMI + 0.299 \times Status + 0.455 \times Sex$

"Status" indicates the state of diabetes mellitus in which a value of 1 is used, whereas "Sex" indicates whether male or female; if male, put 1 in "Sex" [9].

Questionnaire

Physical activity of subjects was assessed using the International Physical Activity Questionnaire short version, whereas the health-related quality of life was assessed using the EuroQOL 5 dimensions 5 level (EQ5D-5L). The QOL scores were calculated from the EQ5D-5L results, as previously described [19]. The history of falls was assessed among subjects who had suffered a fall in the past year. Those who had fallen more than once were counted as having suffered a fall. Exercise habit was defined as exercising at least twice a week for at least 30 minutes each time for at least one year. Sarcopenia was diagnosed according to the diagnostic criteria of the Asia Working Group for Sarcopenia [20], and frailty was screened according to the Japanese version of the frailty diagnostic criteria, which was developed based on the work of Fried et al. [21].

Statistical analysis

Based on the score for the question "Does exercise make you tired?" in the EBBS, which was scored on a five-point Likert scale, with 1 representing "I don't think so at all" and 5 being "I think so absolutely", those who selected 1 or 2 were classified into the no-fatigue group, whereas those who selected 3-5 were classified into the fatigue group.

Statistical analysis was performed using the Shapiro-Wilk test to confirm normality beforehand. The unpaired t-test and Wilcoxon signed-rank sum test were used to analyze normally and nonnormally distributed data, respectively. The frequency data were compared using the chi-square test. For the logistic regression analysis, fatigue was used as the objective variable, and four factors were selected that were likely to influence fatigue using the forced entry method: age, presence of diabetic neuropathy, ACVT, and AEE.

All statistical analyses were conducted using JMP (version 14; SAS Institute Inc.), with the significance level set at 5%.

The effect size, which was calculated using G*Power to discriminate between groups, was estimated using Cohen's d and denoted as d in the results. Values between 0.20-0.49, 0.50-0.79, 0.80-1.29, and >1.30 indicated small, medium, large, and very large effects, respectively. Values <0.20 were considered to indicate no noticeable effect. No missing data was observed in this study.

## Results

All participants (32 males and 18 females) had type 2 diabetes mellitus, with a mean age of 65.3 ± 12.0 years and a mean duration of diabetes of 10 years (range, 3-21 years). Based on fatigue status, 29 patients were assigned to the fatigue group and 21 to the no-fatigue group. Patient characteristics are summarized in Table 1. Compared to the no-fatigue group, the fatigue group was significantly older (p=0.003, CI: -15.5 to -3.7) and had a higher proportion of smokers (p=0.043). Furthermore, significant differences were observed in MNSI scores (p=0.012), QOL scores (p=0.031), and prevalence of diabetic neuropathy (p=0.008).

**Table 1:** Characteristics of patients included in this study The values are presented as means ± standard deviations, medians interquartile ranges (IQRs), or n (%). The unpaired t-test and Wilcoxon signed–rank sum test were used with the significance level set at 5%. MNSI - Michigan Neuropathy Screening Instrument; QOL - quality of life; IPAQ - International Physical Activity Questionnaire

Variable	No Fatigue (n = 21)			Fatigue (n = 29)			P-value	T-score	Chi-squared value	95%CI	Effect size	1-β
Age (years)	70.8 ± 9.1			60.7 ± 12.4			0.003	-3.16		-15.5 to -3.7	0.93	0.89
Sex (male:female)	12:9			20:9			0.391		0.74			
Height (cm)	159.0 ± 9.1			163.0 ± 8.9			0.133	1.53		-1.25 to 9.15	0.44	0.32
BMI (kg/m^2^)	23.8 ± 4.0			26.4 ± 4.8			0.052	1.99		-0.02 to 5.15	0.58	0.51
HbA1c (%)	10.5 ± 1.7			10.2 ± 1.6			0.581	0.68		-1.24 to 0.71	0.18	0.15
Diabetes duration (years)	10 (5-24)			10 (2-15)			0.165	1.39				
Hypertension	11 (52%)			13 (45%)			0.598		0.28			
Dyslipidemia	15 (71%)			17 (59%)			0.349		0.89			
Smoking	7 (33%)			18 (62%)			0.043		4.09			
MNSI	1 (0-2)			3 (2-4)			0.012	-2.52				
QOL score	0.90 (0.83-0.94)			0.82 (0.73-0.90)			0.031	2.16				
Diabetic peripheral neuropathy	9 (42%)			23 (79%)			0.008		7.09			
Diabetic retinopathy	7 (33%)			9 (31%)			0.864		0.03			
Diabetic nephropathy	2 (10%)			1 (3%)			0.375		0.79			
Exercise habits	10 (48%)			7 (24%)			0.084		2.99			
IPAQ	2034 (520-4348.5)			560 (0-4851)			0.322	0.99				
Fall	4 (19%)			7 (24%)			0.666		0.19			

Table 2 shows the factors promoting and inhibiting exercise. The fatigue group reported significantly lower scores for psychological benefit and self-improvement on the exercise benefit scale. Additionally, all five subscales of the exercise barrier scale showed significant differences between the two groups.

**Table 2:** Exercise benefits and barriers scale The values are presented as medians (IQRs). The Wilcoxon signed–rank sum test was used with the significance level set at 5%.

Variables	No fatigue (n = 21)			Fatigue (n = 29)			P-value	T-score
Physical benefit	9 (8-10)			8 (7-10)			0.178	1.35
Psychological benefit	9 (7-10)			8 (6-9)			0.045	2.00
Social benefit	7 (6-8)			6 (4-8)			0.075	1.78
Weight management	8 (5-10)			7 (5-9)			0.376	0.89
Self-improvement	7 (5-9)			5 (4-7)			0.045	2.00
Lack of motivation	2 (2-3)			6 (5-6)			< 0.001	-5.93
Lack of time	3 (2-5)			5 (4-7)			0.045	-2.00
Lack of social support	2 (2-3)			4 (3-6)			0.009	-2.60
Discomfort	3 (2-5)			6 (5-7)			< 0.001	-3.74
Poor physical environment	2 (2-4)			5 (4-6)			0.008	-2.67

As shown in Table 3, there were no significant differences between the two groups in handgrip strength, knee extension force (KEF), walking speed, sarcopenia, frailty, or skeletal muscle mass index (SMI), indicating that physical function and performance were comparable.

**Table 3:** Physical function and performance The values are presented as means ± standard deviations or n (%). The unpaired t-test and the chi-square test were used with the significance level set at 5%. KEF - knee extension force; SMI - skeletal muscle index.

Variable	No fatigue (n = 21)			Fatigue (n = 29)			P-value	T-score	Chi-squared value	95%CI	Effect size	1-β
Hand grip strength (kg)	25.48 ± 10.97			29.65 ± 9.53			0.158	1.43		-1.68 to 10.0	0.41	0.28
KEF (kgf/kg)	0.51 ± 0.16			0.49 ± 0.17			0.636	-0.48		-0.12 to 0.72	0.14	0.08
KEF (kgfm/kg)	0.16 ± 0.05			0.15 ± 0.06			0.869	-0.17		-0.03 to 0.03	0.05	0.05
Walking speed (m/s)	1.24 ± 0.24			1.26 ± 0.24			0.711	0.37		-0.12 to 0.17	0.11	0.07
Sarcopenia	1 (5%)			1 (3%)			0.252		1.31			
Fraility	8 (38%)			13 (45%)			0.852		0.04			
SMI (kg/m^2^)	7.36 ± 1.59			7.63 ± 1.27			0.502	0.68		-0.54 to 1.08	0.19	0.10

**Figure 1:**
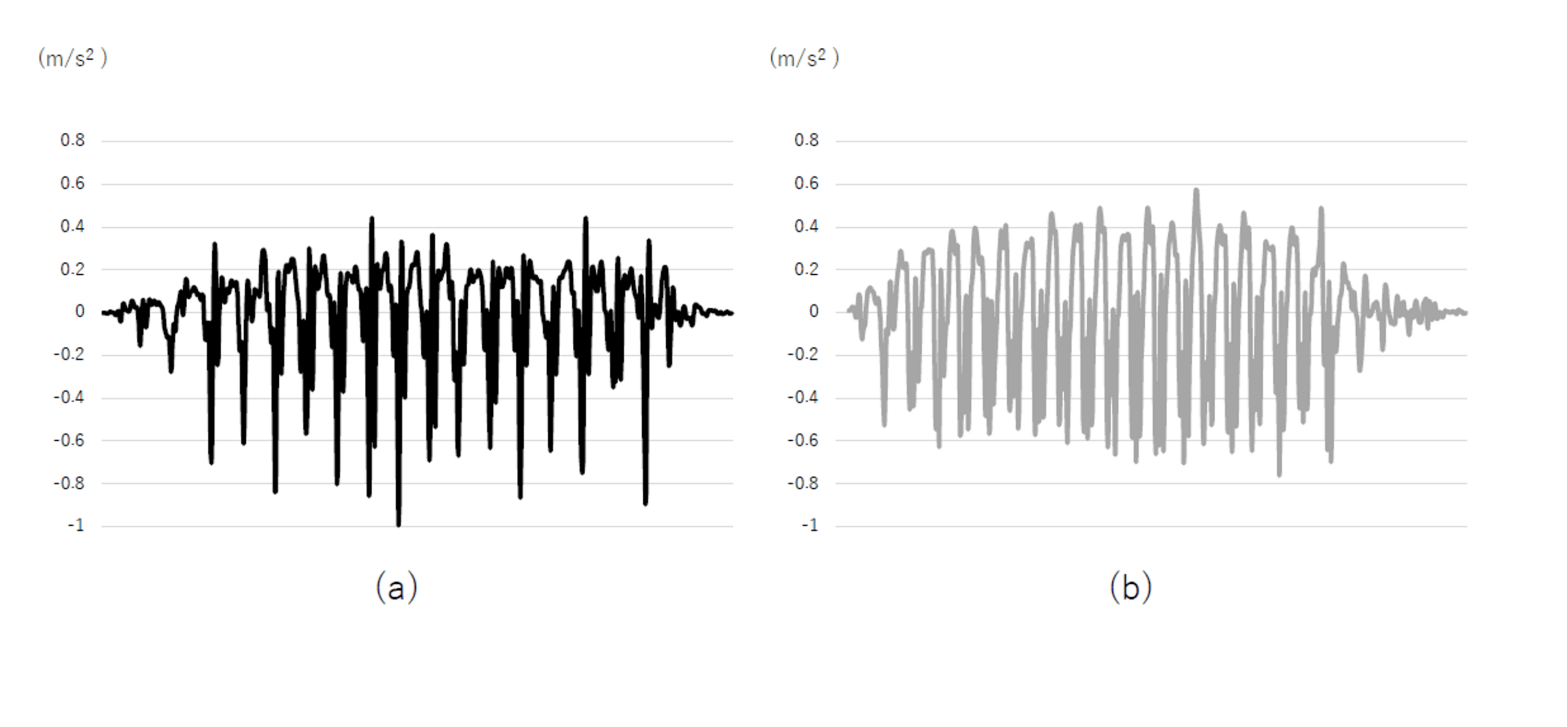
Raw data of acceleration of vertical direction (a) shows a low autocorrelation coefficient because the magnitude of acceleration varies from one walking cycle to the next. On the other hand, (b) shows constant acceleration and a high autocorrelation coefficient.

Accelerometer-based gait parameters are presented in Table 4. No significant differences were found in root mean square (RMS), RMS ratio (RMSR), or harmonic ratio (HR) in any direction. However, as shown in Figure 1, the acceleration coefficient in the vertical direction (ACVT) was significantly lower in the fatigue group (p=0.044, CI: -0.05 to 0.00; effect size=0.60). In addition, activity-related energy expenditure (AEE) was significantly lower in the fatigue group (p=0.039, CI: 0.03 to 0.45; effect size=0.65).

**Table 4:** Acceleration data The values are presented as means ± standard deviations or medians (IQRs). The unpaired t-test and Wilcoxon signed–rank sum test were used with the significance level set at 5%. VT - vertical; ML - medial-lateral; AP - anterior-posterior; RMS - root–mean-square; RMSR - root–mean–square ratio; AC - autocorrelation; HR - harmonic ratio; AEE - activity energy expenditure

Variable	No Fatigue (n = 21)			Fatigue (n = 29)			P-value	T-score	95%CI	Effect size	1-β
RMS_VT_	0.257 ± 0.092			0.286 ± 0.080			0.236	1.20	-0.02 to 0.08	0.34	0.21
RMS_ML_	0.137 (0.116-0.190)			0.164 (0.134-0.262)			0.238	-1.18			
RMS_AP_	0.192 ± 0.062			0.210 ± 0.046			0.258	1.15	-0.01 to 0.05	0.32	0.19
RMSR_VT_	0.700 ± 0.078			0.705 ± 0.059			0.922	0.25	-0.03 to 0.04	0.07	0.06
RMSR_ML_	0.453 ± 0.089			0.459 ± 0.078			0.709	0.24	-0.04 to 0.05	0.07	0.06
RMSR_AP_	0.533 ± 0.084			0.528 ± 0.066			0.990	-0.25	-0.05 to 0.04	0.07	0.06
AC_VT_	0.892 ± 0.034			0.869 ± 0.044			0.044	-2.07	-0.05 to 0.00	0.60	0.50
AC_ML_	0.867 ± 0.063			0.848 ± 0.041			0.196	-1.31	-0.05 to 0.01	0.36	0.24
AC_AP_	0.878 ± 0.045			0.856 ± 0.046			0.098	-1.69	-0.05 to 0.01	0.48	0.38
HR_VT_	2.767 ± 0.723			2.904 ± 0.664			0.494	0.69	-0.26 to 0.53	0.20	0.17
HR_ML_	2.152 ± 0.689			2.013 ± 0.462			0.395	-0.86	-0.47 to 0.19	0.24	0.20
HR_AP_	2.869 ± 0.848			2.916 ± 0.840			0.847	0.19	-0.44 to 0.53	0.06	0.07
AEE (kcal min^-1^)	1.486 ± 0.346			1.724 ± 0.378			0.039	2.27	0.03 to 0.45	0.65	0.61

Finally, Table 5 shows the results of logistic regression analysis. Age (p=0.008, OR=0.916, CI: 0.84 to 0.98) and ACVT (p=0.038, OR=0.16, CI: 0.02 to 0.90) were identified as significant independent predictors of fatigue status. Regarding ACVT, the odds ratio calculated for a 1-unit increase in the explanatory variable yielded an unrealistically large value. Therefore, to improve interpretability and present more meaningful results, the odds ratio was recalculated based on a 0.1-unit increase.

**Table 5:** Logistic regression analysis To avoid overestimation, the odds ratio for ACVT was recalculated based on a 0.1-unit increase instead of a 1-unit increase. AC - autocorrelation; AEE - activity energy expenditure

Variables	P-value	Odds ratio	95％CI
Age (years)	0.008	0.92	0.84 to 0.98
Diabetic peripheral neuropathy	0.167	3.07	0.64 to 18.76
AC_VT_	0.038	0.16	0.02 to 0.90
AEE (kcal min^-1^)	0.117	5.95	0.65 to 80.72

## Discussion

Patients with diabetes who experienced fatigue during exercise had higher MNSI scores, greater barriers to exercise continuity, lower regularity in vertical acceleration, and higher estimated energy expenditure during walking than those who did not. These findings indicate that lower regularity of vertical acceleration during walking and increased AEE are factors causing fatigue.

Although no significant differences in RMS and RMSR were observed between the fatigue and no-fatigue groups, a significant difference was found in AC, which represents gait regularity and is calculated as the correlation of acceleration signals shifted by one gait cycle. The absence of significant differences in RMS may be attributable to the inclusion of participants with only mild sensory impairments. Moreover, since group classification was based on subjective fatigue status rather than the severity of DPN, this may have further reduced the likelihood of detecting significant differences in RMS parameters. Previous studies have suggested that maintaining a consistent walking pace with rhythmic stimulation can reduce both energy expenditure and fatigue [22]. The observed increase in AEE, calculated using acceleration data, body mass index, and sex, indicates elevated oxygen consumption [9], which has been shown to correlate positively with fatigue [23]. Therefore, the low AC in the fatigue group, which reflects lower gait regularity, may have contributed to inefficient walking patterns, thereby increasing oxygen consumption and ultimately leading to greater fatigue. Based on these findings, we suggest that reduced acceleration regularity during gait, particularly in the vertical direction, is a characteristic feature associated with fatigue during exercise in patients with type 2 diabetes.

Several studies investigating experimentally induced fatigue changes in gait dynamics have reported that fatigue affects gait parameters, such as the RMS of trunk acceleration [14]. However, these studies observed walking during extreme fatigue due to the exercise load. Considering that our participants did not achieve a fatigued state, no differences in RMS and RMSR were observed between the two groups. Reports have shown that HR is associated with the risk of falling [24]; however, it is not influenced by age or walking speed [25]. Thus, fatigue had little effect on HR.

This study showed that the fatigue group exhibited higher DPN rates than the no-fatigue group. DPN has been considered a cause of muscle fatigue due to the heterogeneity of motor units and motor neuropathy [26]. Therefore, DPN may be a factor that predisposes patients with diabetes to fatigue during exercise. Although previous studies have suggested a link between DPN and increased muscle fatigue due to altered motor unit recruitment, others argue that subjective malaise does not always align with objective fatigue indicators in DPN patients. This discrepancy warrants further investigation [5]. On the other hand, previous studies have reported that DPN can affect vertical acceleration during walking [27]. Based on these findings, we suggest that differences in gait patterns, such as lower regularity in vertical acceleration, may contribute to fatigue in patients with DPN. However, as the underlying mechanisms through which DPN influences gait patterns remain unclear, further investigations are needed, with a focus on vertical acceleration.

Notably, although no significant differences in physical function were observed between the two groups, significant differences in gait parameters were observed. This may be affected by age and MNSI scores (i.e., the fatigue group was younger but had higher MNSI scores), factors that may affect physical function.

Although no significant differences in exercise habits were observed (p=0.084), the fatigue group had lower values (24%) than the no-fatigue group (48%) (Table 1). The Nutrition Examination Survey (2018) by the Ministry of Health, Labor, and Welfare of Japan [28] reported that 31.8% of Japanese men and 25.5% of Japanese women have exercise habits, suggesting that the fatigue (24%) and no-fatigue (48%) groups have similar or greater exercise habits than the general Japanese population. Therefore, fatigue during exercise in patients with diabetes should be considered to be potentially influenced by gait patterns and exercise regularity rather than physical function.

Studies on the general elderly population have reported that fatigue due to walking increases the risk of falls [29]. Furthermore, an intervention study involving patients with type 2 diabetes reported that the combination of aerobic exercise and resistance training increases muscle strength and QOL and reduces fatigue [30]. However, a study on patients with multiple sclerosis reported the detection of gait dynamics and fatigue using a wearable inertial sensor [31]. As a method of intervention for elderly patients with diabetes (expected to increase in number worldwide in the future), using exercise therapy, including aerobic exercise and resistance training, and modification of gait patterns may be necessary as an approach to reduce fatigue during exercise and increase exercise continuity. This is beneficial for both clinicians and patients. Future research should examine the content of interventions for gait and the effects of gait modification on fatigue.

This study has some limitations. First, the present study measured only subjective fatigue, so it did not measure multifaceted fatigue and exercise tolerance, such as the exhaled gas analyzer or the six-minute walk test. In the future, other factors influencing fatigability, including exercise tolerance, should be assessed. Additionally, regarding AEE, this study used an accelerometer rather than a breath gas analyzer for calculation, which poses accuracy issues. However, previous studies targeting diabetic patients have shown a high coefficient of determination (R²=0.81-0.85) between accelerometer-estimated values and indirect calorimetry [9]. Considering these results and clinical feasibility, this study adopted the accelerometer method for estimating AEE. Second, gait characteristics other than acceleration were not measured. The gold standard for motion analysis is three-dimensional motion analysis using an infrared camera, which is highly accurate and reliable but expensive. However, a previous study showed that for gait parameters, the values of the three-dimensional motion analyzer and accelerometer exhibited moderate to strong correlations, ranging from 0.6 to 0.9 [32]. Furthermore, measurements using an accelerometer, particularly a smartphone, can be easy to perform in clinical situations, and this method is expected to have applications in the future. Third, although an a priori sample size calculation was not performed in this study, we conducted a post hoc power analysis to justify the adequacy of the sample size with respect to the anticipated effect size. Using G*Power, a hypothetical calculation assuming an effect size of 0.8, a significance level α of 0.05, and a power (1-β) of 0.8 indicated that a minimum of 21 participants would be required. Given that our study included 21 and 29 participants, we consider the sample size sufficient to detect a medium to large effect. Fourth, in this study, it was not possible to completely control for confounding factors such as complications other than diabetes. This may have affected the results of the study. It is therefore essential that future research designs incorporate strategies to control for these variables to enhance internal validity and the robustness of conclusions. Finally, this study had certain real-time limitations related to COVID-19, which may have influenced the generalizability and completeness of data. These factors should be considered when interpreting the results.

## Conclusions

In this study, logistic regression analysis identified the regularity of vertical acceleration during walking as an independent factor associated with the presence or absence of subjective fatigue. This finding suggests that reduced regularity in gait may contribute to the experience of fatigue, potentially acting as a barrier to exercise continuity. However, given the cross-sectional design of the study, these results should be interpreted with caution. Future research is needed to further investigate the underlying mechanisms through which gait irregularity leads to fatigue, and to evaluate the effectiveness of interventions aimed at improving gait patterns, with the goal of reducing walking-related fatigue and overcoming barriers to regular exercise.

## References

[1] Yimer YS, Addissie A, Kidane EG, Reja A, Abdela AA, Ahmed AA (2025). Effectiveness of diabetes self-management education and support interventions on glycemic levels among people living with type 2 diabetes in the WHO African Region: a systematic review and meta-analysis. Front Clin Diabetes Healthc.

[2] Sato Y, Sone H, Kobayashi M (2015). Current situation of exercise therapy in patients with diabetes mellitus in Japan (report no. 2): a nationwide survey to patients using the questionnaires (Article in Japanese). J Jpn Diabetes Soc.

[3] Fritschi C, Quinn L (2010). Fatigue in patients with diabetes: a review. J Psychosom Res.

[4] Gandevia SC (2001). Spinal and supraspinal factors in human muscle fatigue. Physiol Rev.

[5] Orlando G, Sacchetti M, D'Errico V, Haxhi J, Rapisarda G, Pugliese G, Balducci S (2020). Muscle fatigability in patients with type 2 diabetes: relation with long-term complications. Diabetes Metab Res Rev.

[6] Mustapa A, Justine M, Mohd Mustafah N, Jamil N, Manaf H (2016). Postural control and gait performance in the diabetic peripheral neuropathy: a systematic review. Biomed Res Int.

[7] Cronin NJ, Peltonen J, Ishikawa M, Komi PV, Avela J, Sinkjaer T, Voigt M (2010). Achilles tendon length changes during walking in long-term diabetes patients. Clin Biomech (Bristol).

[8] Brown SJ, Handsaker JC, Bowling FL, Maganaris CN, Boulton AJ, Reeves ND (2014). Do patients with diabetic neuropathy use a higher proportion of their maximum strength when walking?. J Biomech.

[9] Caron N, Peyrot N, Caderby T, Verkindt C, Dalleau G (2020). Estimating energy expenditure from accelerometer data in healthy adults and patients with type 2 diabetes. Exp Gerontol.

[10] Caron N, Peyrot N, Caderby T, Verkindt C, Dalleau G (2018). Effect of type 2 diabetes on energy cost and preferred speed of walking. Eur J Appl Physiol.

[11] Heise GD, Martin PE (2001). Are variations in running economy in humans associated with ground reaction force characteristics?. Eur J Appl Physiol.

[12] Rabuffetti M, Steinach M, Lichti J (2021). The Association of fatigue with decreasing regularity of locomotion during an incremental test in trained and untrained healthy adults. Front Bioeng Biotechnol.

[13] Garcia RE, Blackwell TL, Forman DE (2024). Role of walking energetics and perceived fatigability differs by gait speed: the study of muscle, mobility and Aging (SOMMA). J Gerontol A Biol Sci Med Sci.

[14] Schütte KH, Maas EA, Exadaktylos V, Berckmans D, Venter RE, Vanwanseele B (2015). Wireless tri-axial trunk accelerometry detects deviations in dynamic center of mass motion due to running-induced fatigue. PLoS One.

[15] Ishii K, Inoue S, Ohya Y (2009). Development of a short version of the perceived benefits and barriers to exercise scale (Article in Japanese). Jpn J Phys Fit Sports Med.

[16] Nomura T, Ishiguro T, Ohira M, Ikeda Y (2018). Diabetic polyneuropathy is a risk factor for decline of lower extremity strength in patients with type 2 diabetes. J Diabetes Investig.

[17] Moghtaderi A, Bakhshipour A, Rashidi H (2006). Validation of Michigan neuropathy screening instrument for diabetic peripheral neuropathy. Clin Neurol Neurosurg.

[18] Speedtsberg MB, Christensen SB, Stenum J, Kallemose T, Bencke J, Curtis DJ, Jensen BR (2018). Local dynamic stability during treadmill walking can detect children with developmental coordination disorder. Gait Posture.

[19] Shiroiwa T, Ikeda S, Noto S, Igarashi A, Fukuda T, Saito S, Shimozuma K (2016). Comparison of value set based on DCE and/or TTO data: scoring for EQ-5D-5L health states in Japan. Value Health.

[20] Chen LK, Woo J, Assantachai P (2020). Asian Working Group for sarcopenia: 2019 consensus update on Sarcopenia diagnosis and treatment. J Am Med Dir Assoc.

[21] Satake S, Arai H (2020). The revised Japanese version of the Cardiovascular Health Study criteria (revised J-CHS criteria). Geriatr Gerontol Int.

[22] Fattorini L, Rodio A (2019). Acoustic and visual pacesetter influence on the energy expenditure in a cycling exercise. J Sports Med Phys Fitness.

[23] Schrack JA, Wanigatunga AA, Zipunnikov V, Kuo PL, Simonsick EM, Ferrucci L (2020). Longitudinal association between energy regulation and fatigability in mid-to-late life. J Gerontol A Biol Sci Med Sci.

[24] Doi T, Hirata S, Ono R, Tsutsumimoto K, Misu S, Ando H (2013). The harmonic ratio of trunk acceleration predicts falling among older people: results of a 1-year prospective study. J Neuroeng Rehabil.

[25] Lowry KA, Lokenvitz N, Smiley-Oyen AL (2012). Age- and speed-related differences in harmonic ratios during walking. Gait Posture.

[26] Watanabe K, Miyamoto T, Tanaka Y, Fukuda K, Moritani T (2012). Type 2 diabetes mellitus patients manifest characteristic spatial EMG potential distribution pattern during sustained isometric contraction. Diabetes Res Clin Pract.

[27] Eatough ZJ, Peterson AC, Lisonbee RJ (2024). Static posture weightbearing joint angle differences in patients with varus ankle osteoarthritis. Gait Posture.

[28] (2018). MHLW: National health and nutrition survey. https://wwwmhlwgojp/toukei/itiran/gaiyo/k-eiseihtml.

[29] Morrison S, Colberg SR, Parson HK (2016). Walking-induced fatigue leads to increased falls risk in older adults. J Am Med Dir Assoc.

[30] Tomas-Carus P, Ortega-Alonso A, Pietilainen KH (2016). A randomized controlled trial on the effects of combined aerobic-resistance exercise on muscle strength and fatigue, glycemic control and health-related quality of life of type 2 diabetes patients.. J Sports Med Phys Fit.

[31] Müller R, Hamacher D, Hansen S, Oschmann P, Keune PM (2021). Wearable inertial sensors are highly sensitive in the detection of gait disturbances and fatigue at early stages of multiple sclerosis. BMC Neurol.

[32] Furrer M, Bichsel L, Niederer M, Baur H, Schmid S (2015). Validation of a smartphone-based measurement tool for the quantification of level walking. Gait Posture.

